# Ultra-Long-Term CT Angiography Evaluation of Patients Treated with Covered Stents for Visceral Aneurysms: A Single Center Case Series

**DOI:** 10.3390/diagnostics15121481

**Published:** 2025-06-11

**Authors:** Marcello Andrea Tipaldi, Nicolò Ubaldi, Edoardo Ronconi, Michela Ortenzi, Francesco Arbia, Gianluigi Orgera, Miltiadis Krokidis, Tommaso Rossi, Pasqualino Sirignano, Luigi Rizzo, Michele Rossi

**Affiliations:** 1 Interventional Radiology Unit, Department of Surgical and Medical Sciences and Translational Medicine, Sant’Andrea Hospital of Rome, “Sapienza” University of Rome, 00189 Rome, Italy; tipaldi.andrea@gmail.com (M.A.T.); edoardo.roncon1@gmail.com (E.R.); michelaortenzi16@gmail.com (M.O.); francescoarbia@hotmail.it (F.A.); orgera.g@gmail.com (G.O.); michele.rossi@uniroma1.it (M.R.); 2 School of Medicine, Areteion Hospital, National and Kapodistrian University of Athens, 11528 Athens, Greece; mkrokidis@hotmail.com; 3 Interventional Radiology Unit, Department of Diagnostic Medicine and Radiology, Policlinico Umberto I Hospital of Rome, “Sapienza” University of Rome, 00161 Rome, Italy; tommirossi97@hotmail.it; 4 Vascular and Endovascular Surgery Unit, Department of General and Specialistic Surgery, Sant’Andrea Hospital of Rome, “Sapienza” University of Rome, 00189 Rome, Italy; pasqualino.sirignano@uniroma1.it; 5 Vascular and Endovascular Surgery Unit, Department of Clinical and Molecular Medicine, Sant’Andrea Hospital of Rome, “Sapienza” University of Rome, 00189 Rome, Italy; luigi.rizzo@uniroma1.it

**Keywords:** CT angiography, covered stent, stent graft, visceral aneurysm, pseudoaneurysm, endovascular

## Abstract

**Objective:** Endovascular repair of visceral artery aneurysms (VAAs) and visceral artery pseudoaneurysms (VAPAs) using covered stent grafts is a novel technique that preserves efferent vessel patency and prevents end-organ ischemia; however, long-term results are lacking in the literature. This study aims to evaluate ultra-long-term outcomes (>5 years) using CT angiography (CTA) and technical aspects of covered stents in treating VAAs and VAPAs. **Methods:** A single-center retrospective study was conducted on patients with VAAs and VAPAs treated with stent grafts between 2004 and 2023. The study included an ultra-long-term follow-up using CTA. Stent graft patency, aneurysm characteristics, technical success, 30-day and long-term follow-up clinical success, and mortality were assessed. **Results:** Among 23 patients presenting with VAAs and VAPAs treated exclusively with covered stents implantation, 7 (mean age: 68 years, SD 14), including 5 with VAAs and 2 with VAPAs, met the inclusion criteria for the study. Six of the seven patients underwent elective procedures with no significant periprocedural complications. Both technical and 30-day clinical success rates were 100%. The mean follow-up period was 10 years (125 months SD 53). At the 5-year follow-up, 71% of stent grafts remained patent. No patient experienced aneurysm sac revascularization or rupture. Stent obstruction did not affect survival. **Conclusions:** This study demonstrates that endovascular covered stenting is a durable and effective treatment for VAAs and VAPAs, even in the ultra-long term, with a patency rate of 71% at a mean CTA follow-up of 125 months, the longest reported to date and no cases of sac revascularization. Stent thrombosis was significantly associated with VAPAs.

## 1. Introduction

Visceral artery aneurysms (VAAs) and visceral artery pseudoaneurysms (VAPAs) are rare but potentially life-threatening vascular conditions, with an incidence ranging from 0.01% to 0.2% [1]. In recent years, their incidence has risen, particularly in Western countries, due to an increase in vascular pathologies [2]. Additionally, the widespread use of cross-sectional abdominal imaging, such as computed tomography angiography (CTA) and magnetic resonance imaging (MRI), has contributed to a higher detection rate [3].

VAAs typically arise from arteriosclerotic damage and medial degeneration of blood vessels, which is more common in older patients. In younger patients, fibromuscular dysplasia can sometimes be the underlying cause [4]. VAPAs are often caused by inflammation (e.g., pancreatitis or cholecystitis), infection, dissection, or vascular trauma, including iatrogenic factors [4,5,6]. Diagnosis of a VAA is frequently incidental, detected during contrast-enhanced abdominal imaging, most commonly CT, which is also useful for localization, characterization, and treatment planning [7]. While VAAs are often small and asymptomatic, their presentation may vary. Splenic artery aneurysms are the most common type of VAA, accounting for approximately 60% of cases, followed by hepatic (20–50%), superior mesenteric (6%), and celiac artery aneurysms (4%) [4]. Although most VAAs remain stable in size, certain conditions, such as pregnancy or hypertension, or the presence of infection, can increase the risk of rupture [8]. In contrast, VAPAs tend to progress more rapidly, with rupture occurring in 76.3% of cases, compared to only 3.1% for VAAs [9]. The mortality rate for VAPAs is alarmingly high, reaching up to 76% [4].

Indications for treating true aneurysms typically depend on their location and size, while pseudoaneurysms should be treated regardless of size [10,11]. Treatment options include both surgical and endovascular approaches. Due to its reduced invasiveness and lower morbidity compared to surgery, as well as advancements in technical materials and expertise, the endovascular approach has become the preferred treatment for the vast majority of VAAs in many centers [12,13,14]. The choice of treatment technique depends largely on the interventional radiologist’s experience and the specific anatomy of the aneurysm [15]. Endovascular options include transcatheter embolization using coils, glue, liquid embolic agents, or plugs, stent grafts, percutaneous thrombin injection (for superficial aneurysms), or a combination of these techniques. The use of covered stent grafts in the treatment of VAAs and VAPAs offers the advantage of aneurysm exclusion while preserving parent vessel flow, reducing the risk of distal ischemia seen with transcatheter embolization [16]. However, this approach can be limited by the tortuosity of visceral arteries [5,8] or unfavorable aneurysm anatomy. Despite its growing popularity, the long-term patency of covered stents remains unclear. Few studies have examined stent graft patency for aneurysm exclusion in the mid-term [16,17], and none have reported follow-up periods longer than five years.

The aim of this retrospective study is to evaluate the ultra-long-term outcome of covered stents, defined as a follow-up period of at least five years using CT angiography.

## 2. Materials and Methods

### 2.1. Study Design

This is a retrospective single-center study including patients who underwent endovascular repair of a VAA or a VAPA with a covered stent graft between 2004 and 2023. However, only patients treated before 2018 were eligible for final analysis to ensure a minimum follow-up of 5 years. Inclusion criteria were the following: (i) treatment of a VAA or a VAPA with endovascular repair, (ii) use of a balloon mounted or self-expandable stent graft without other endovascular tools, and (iii) imaging follow-up with contrast-enhanced CT scan of at least five years. Exclusion criteria were the following: (i) treatment of a VAA or a VAPA with other endovascular tools, and (ii) clinical and imaging follow-up less than five years.

According to the local protocol and to CIRSE guidelines [10], patients underwent treatment in the following cases: (a) symptomatic aneurysm/pseudoaneurysm, (b) asymptomatic aneurysm with >2 cm, and (c) pseudoaneurysm regardless of their size. Growth rate of >0.5 mm per year was an indication for treatment for patients under surveillance; however, patients only when the lesion reached 2 cm in diameter. Clinical information (chronic visceral inflammation or post-traumatic) and CT imaging (morphology, location, and enhancement) were collected in all cases.

Written informed consent was obtained from all patients before treatment. Consent for data use was acquired for all patients after 2013, and for those treated earlier, it was obtained retrospectively during follow-up. This study was conducted in accordance with the guidelines of the Declaration of Helsinki, and approval from the local ethics committee was obtained.

### 2.2. Follow-Up and CT Protocol

Follow-up included clinical examinations and/or imaging tests, using ultrasound or contrast-enhanced CT scans. A CTA scan was performed in all cases before discharge to serve as the baseline examination. Additional contrast-enhanced arterial phase CT scans were performed at 1, 6, and 12 months post-procedure, and subsequently every two years for up to 12 years, provided no re-intervention was necessary.

The CT study was performed using a 256-slice CT (Brilliance iCT 256, Philips Healthcare, Eindhoven, The Netherlands), with a tube voltage of 120 kVp. All CT examinations included a pre-enhanced phase, an angiographic arterial phase, and a venous phase. The acquired native images were evaluated on a workstation with multiplanar reconstructions and maximum intensity projection. Measurements of the aneurysm and afferent and efferent vessels were performed using digital subtraction angiography.

Annual clinical and ultrasound examinations were conducted throughout the follow-up period. If no complications were observed after 12 years, the patient was discharged from further follow-up.

### 2.3. Population

**Figure 1:**
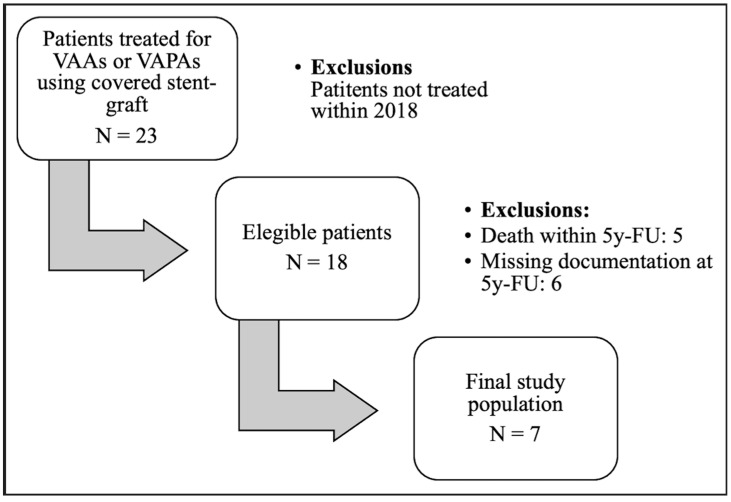
Study population selection flowchart.

A total of 23 patients underwent treatment with covered stents for VAAs or VAPAs at our center between 2004 and 2023. Of these, 18 patients who received treatment before 2018 were initially included in the study population. Eleven patients passed away before completing the five-year follow-up, resulting in a final analyzed population of seven patients. The cohort consisted of 3 males and 4 females, with a mean age of 67 years (Figure 1).

The treated lesions included 5 VAAs and 2 VAPAs. The aneurysms were located in the common hepatic artery (*n* = 1), splenic artery (*n* = 5), and renal artery (*n* = 1). Six patients were treated electively, and one was treated in an emergency setting (Table 1). VAPAs were located in the common hepatic artery and splenic artery.

### 2.4. Procedural Technique

A CT angiogram was performed for all patients prior to treatment. The decision to use a stent graft was based on factors such as access route, vessel angulation, and tortuosity, as well as the location and size of the aneurysm or pseudoaneurysm. All procedures were performed by experienced interventional radiologists, each with over 5 years of experience.

The procedures were conducted under local anesthesia with light sedation in the angiography suite. The preferred access route was the right common femoral artery, particularly when a larger sheath was required. Various catheters and guidewires were used based on the operator’s preference. Covered stents used in the procedures included self-expanding stents such as the Viabahn (W.L. Gore & Associates, Flagstaff, AZ, USA) and the Symbiot (Boston Scientific Corporation, Marlborough, MA, USA), as well as balloon-expandable stents such as the Jostent (Abbott Vascular, Rangendingen, Germany) (Table 1). Stent size was chosen according to the main vessel diameter, 10–20% oversized depending on if it was a VAPA or VAA, respectively. If the aneurysm was not completely excluded, a second stent was placed, partially overlapping the first. Balloon remodeling was performed when necessary to better conform the stent graft to the arterial wall. After stent placement, and only in hemodynamically stable patients, 5000 units of heparin were infused intra-arterially. Prophylactic antibiotics were administered post-deployment.

Elective patients received aspirin (100 mg/day) for at least five days prior to the procedure. Post-procedure, dual antiplatelet therapy (aspirin 100 mg/day and clopidogrel 75 mg/day) was continued for one month, followed by lifelong aspirin therapy (100 mg/day).

### 2.5. Outcomes Measures

The primary outcome was stent graft patency and the continued exclusion of the aneurysm sac, evaluated from five years post-treatment using follow-up contrast-enhanced CT scans. Stent patency was assessed based on in-stent lumen visibility and distal runoff opacification. Patients were categorized into two groups: those with patent stents and those with stent occlusion.

Secondary outcomes included immediate technical success and 30-day clinical success, as well as periprocedural and long-term complications. Technical success was defined as the complete exclusion of the aneurysm, confirmed by final angiographic assessment. Thirty-day clinical success was defined as the resolution of signs and symptoms related to the aneurysm, based on both imaging and clinical data at one month.

Complications were classified as minor or major (including extended hospitalization, permanent adverse effects, or mortality), following international standard guidelines [18].

### 2.6. Statistical Analysis

Comparative analyses were executed using the χ^2^ test and Fisher’s exact test, contingent upon the data. IBM SPSS Statistics for Windows, Version 25, was the tool of choice for statistical analysis. Continuous variables were articulated as means ± standard deviation, while categorical variables were represented as percentages. A *p*-value of ≤0.05 was the threshold for statistical significance.

## 3. Results

All lesions were successfully treated with a technical success rate and clinical success at 30 days for both VAAs and VAPAs of 100%. The mean follow-up period was 125 months (range 60–190; SD 53). Technical features of the procedure are documented in Table 2.

At the 5-year mark post-treatment, five out of seven stent grafts (71%) were documented as patent (Table 3). All of the patients had complete aneurysm exclusion without revascularization.

Regarding the patient cohort with stent obstruction at 5 years, the stent grafts were utilized to treat one splenic pseudoaneurysm in emergency and one aneurysm in election, both thrombosed at 6 months. Both presented extravascular migration (29%) (Table 3); the first became untraceable, whilst the second migrated in the lesser omentum.

**Figure 2:**
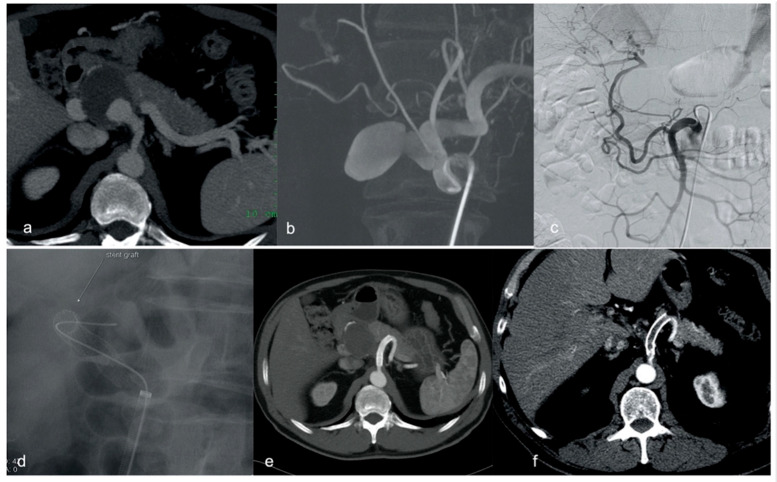
Multimodal imaging and long-term follow-up of a hepatic pseudoaneurysm. (**a**) Angio CT shows a pseudoaneurysm of the hepatic artery origin with dissection and obstruction of the artery; (**b**) dynamic CT angiography used for pre-treatment planning; (**c**) angiogram of the superior mesenteric artery demonstrating collateral flow to the distal hepatic artery; (**d**) intraoperative image of Viabahn stent graft deployment in the coeliac trunk and in the splenic artery to exclude the aneurismatic origin of the hepatic artery; (**e**) CT at 5 years with patency of the implanted stent graft; (**f**) occluded stent graft at 139 months follow-up.

A third patient experienced stent graft obstruction (Table 3), used for an elective pseudoaneurysm lesion repair, which occurred 139 months after the procedure. This specific patient presented to the Emergency Department with diffused abdominal pain and the CT scan documented stent thrombosis with proximal dislocation and mild splenic infarcts, three months after quitting the aspirin administration given for maintenance (Figure 2).

There was no significant difference in terms age or average size of the aneurysm between those with obstructed and patent stents, *p*-value = 0.5924 and *p*-value = 0.5239, respectively. There were also no significant differences in terms of the shape of the lesion and location (Table 3). Overall, no patient manifested aneurysm sac revascularization nor rupture throughout the follow-up period.

## 4. Discussion

The present study reinforces the role of endovascular stent grafting as a durable and effective treatment for both visceral artery aneurysms and pseudoaneurysms, with encouraging results even in the long term. While previous literature predominantly focuses on short- to mid-term follow-up (2–3 years) [5,8,16,17], our findings provide valuable insights extending beyond 10 years—a timeframe rarely reported in the existing literature due to the inherent challenges of patient retention and mortality in this population. Despite the small sample size, consistency in follow-up and outcome evaluation enhances the clinical relevance of our findings.

Stent patency at 5 years was achieved in 71% of patients, suggesting good mid-term durability, and this adds novel information about ultra-long-term outcomes. Interestingly, two of the three observed stent occlusions occurred early, within 6 months of treatment, while the third occurred very late (139 months), suggesting the importance of long-term CTA follow-up schedules.

Visceral artery aneurysms and pseudoaneurysms are infrequent yet serious vascular conditions, with an estimated incidence rate of up to 0.2% and a rupture risk of approximately 25–40%, associated with a mortality rate as high as 76% [1,4].

The management consists of two main types of treatment: open repair and endovascular repair. The improvement of endovascular techniques is leading to a significant decrease in the use of the open one, especially due to observed lower perioperative complications, morbidity, and mortality of the former [19]. There are several factors to consider when choosing the correct endovascular approach, such as the aneurysm site, size, shape, caliber of the aneurysm neck, and the number and characteristics of the feeding arteries [20]. The contrast-enhanced CT scan proved valuable not only in the identification and monitoring of the lesions, but also in providing essential technical information for guiding the procedural approach. This information included determining the optimal arterial access according to the anatomy, sizing, and length of the stent graft, and identifying efferent vessels that require embolization. Overcoming the challenges posed by the marked tortuosity and typically small diameter of the visceral arteries is often the primary problem when considering stent graft feasibility.

The introduction of stent grafts in the endovascular treatment of aneurysmal lesions currently contributes to the conservative treatment of even large-neck and fusiform aneurysms, with the opportunity of sac exclusion and efferent arteries’ patency preservation in 90–95% of cases evaluated in the short-term [21], allowing preservation of systemic flow to the end organ. Low-profile, reasonable flexibility, and adaptability to various artery diameters and aneurysm lengths are the advantageous features of this device.

The main limitation of stent deployment technique is the low feasibility in case of elevated tortuosity and narrowed caliber of visceral arteries. Several crucial technical attentions are necessary to successfully treat VAAs and VAPAs using a stent graft. These include the following: (a) necessity for a stable carrier system to safely advance the stent graft; (b) large enough sheath to contain the stent system; (c) selection of a stent graft with suitable dimensions and structural characteristics-flexibility; (d) deploying the stent correctly proximally and distally to the aneurysm.

In our center, self-expandable covered stents are generally preferred to balloon-expandable ones for the treatment of VAAs and VAPAs, mainly due to the inherent tortuosity of visceral arteries. Specifically, the self-expandable stent grafts, although less precise in their deployment with regard to the proximal and distal landing sites, are still highly adaptable, lack shape memory, and are more flexible than balloon-expandable stent grafts. The latter are stiffer and better suited for short and straight arteries, as they appear to impose a structural change on the artery, causing it to deviate from its tortuous course and potentially leading an increased risk of thrombosis. An alternative for the treatment of distally located VAAs in small arteries is the use of coronary covered stent grafts, which are more flexible and have a lower profile [5]. A recent option for VAA treatment could be the use of flow-diverting stents, initially invented for cerebral aneurysms, which induce sac thrombosis since the blood flow into the aneurysm reduces its speed and becomes stagnant. This event appears to support aneurysm shrinkage and stabilization, maintaining patent the efferent vessels arising from the sac at the same time. The principal critical factors regarding flow-diverter stents’ use in VAAs treatment deal with the available diameter, their much higher cost, and the longer time required to achieve aneurysm exclusion [22,23].

Emergency stent graft placement was performed in just one patient within the study population experiencing pseudoaneurysm rupture, notably involving an extraordinary instance of stent graft obstruction and subsequent extravascular migration. Specifically, a Viabahn stent graft was deployed in a patient who developed splenic artery pseudoaneurysm rupture after major surgery for neuroendocrine pancreatic tumor. In this case, the follow-up contrast-enhanced CT revealed stent obstruction 6 months after the procedure, with the last stent visualization reported at 4 years using Endoscopic Ultrasonography within the stomach. At the following CT examination, performed 5 years after stent placement, the stent graft was not found. Stent migration was also observed in another patient into the lesser sac at 60 months (Table 2). All covered stents deployed for pseudoaneurysm repair (*n* = 2) were thrombosed within the follow-up period, underlining a potential association between stent thrombosis and pseudoaneurysm with respect to a true aneurysm, although the population is too small to draw any significant conclusions. This could be due to the fragility and irregularity of the single layer wall together with the local inflammation, which increases flow turbulence, blood stagnation, and clotting. This circumstance may lead to migration and potentially result in fistulation with adjacent organs. While no definitive association can be drawn, such findings warrant further investigation in larger patient populations.

The migration of devices, such as stent grafts or coils, employed in endovascular exclusion of aneurysms and pseudoaneurysms is a rare and poorly described complication, potentially both immediate and delayed. Extravascular migration seems to be infrequent. According to the hypothesis formulated in other articles, the coexistence of a recent pancreatic surgery [24], which can lead to vessel and other tissue damage, the inflammatory state facilitated by the neoplastic condition, and the ruptured aneurysm, are factors that may contribute to the stent extravascular migration. Importantly, in our population, there were no deaths directly associated with stent obstruction, as survival was not significantly affected by stent obstruction or migration.

In conclusion, our research adds to the existing literature by analyzing both the covered stent patency rate and aneurysm sac exclusion over an extended mean FU period of more the 10 years, higher than any other research paper to the best of our knowledge [8,22]. The high technical success rates align with findings reported in previous studies [5,8].

### Limitations

This study has limitations, including the small number of cases and the retrospective nature. Given the limited cohort of seven patients, the findings of this case series should be interpreted with caution and may not be generalizable to the wider patient population. However, the rarity of the pathology and the urgent nature of some treatments make it challenging to design prospective trials.

## 5. Conclusions

This retrospective single-center study demonstrates that covered stent grafts represent a safe and effective long-term treatment option for VAAs and VAPAs, achieving high technical and clinical success rates with sustained aneurysm exclusion. At the five-year follow-up, stent patency was observed in 71% of patients, with no cases of aneurysm rupture or revascularization throughout the ultra-long-term CTA follow-up period (up to 190 months, mean 125 months). While stent occlusion did occur in a minority of cases, and both stents for VAPAs did thrombose, it was not associated with life-threatening complications, and in one instance was linked to discontinuation of antiplatelet therapy. These findings underscore the importance of patient adherence to long-term pharmacologic management and regular CTA surveillance. Larger multicenter studies are warranted to validate these results and to further clarify predictors of long-term stent patency.

## Figures and Tables

**Table 1:** Features of the population, the lesions and the stent grafts that were included in the study.

Patient	Sex	Age	Type of Lesion	Location	Setting	Size (mm)	Type of Stent
1	Female	55	VAA	Splenic	Elective	25	Jostent 5 × 38 mm
2	Female	52	VAA	Splenic	Elective	20	Symbiot 5 × 30 mm
3	Male	63	VAPA	Hepatic	Elective	42	Viabahn 8 × 50 mm
4	Male	27	VAA	Renal	Elective	25	Jostent 5 × 19 mm
5	Female	69	VAA	Splenic	Elective	25	Viabahn 6 × 48 mm
6	Female	45	VAPA	Splenic	Emergency	34	Viabahn 5 × 50 mm
7	Male	65	VAA	Splenic	Elective	72	Viabahn 10 × 100 mm

VAA: visceral artery aneurysms; VAPA: visceral artery pseudoaneurysms.

**Table 2:** Procedural characteristics for aneurysm treatment.

Patient	Vascular Access	Sheath	Procedural Time	Aneurysm Configuration	Aneurysm Location	Anaesthesia	Vascular Access
1	Femoral	8F	90 min	Sacciform large neck	Proximal	Local	Femoral
2	Brachial	6F	120min	Sacciform short neck	Mid-way	Local	Brachial
3	Femoral	8F	100 min	Sacciform large neck	Proximal	Local	Femoral
4	Femoral	6F	80 min	Sacciform large neck	Distal	Local	Femoral
5	Brachial	6F	40 min	Sacciform large neck	Mid-way	Local	Brachial
6	Femoral	6F	80 min	Fusiform	Mid-way	Local	Femoral
7	Femoral	8F	90 min	Sacciform large neck	Proximal	Local	Femoral

**Table 3:** Follow-up data for patients included in present series.

Patient	Stent	FU-Duration (Months)	Stent-Graft Patency at 5 Years	Stent Occlusion (Months)	Stent Migration (Months)
1	Jostent 5 × 38 mm	191	Yes	-	-
2	Symbiot 5 × 30 mm	102	No	6	102
3	Viabahn 8 × 50 mm	139	Yes	139	-
4	Jostent 5 × 19 mm	90	Yes	-	-
5	Viabahn 6 × 48 mm	169	Yes	-	
6	Viabahn 5 × 50 mm	79	No	6	60
7	Viabahn 10 × 100 mm	60	Yes	-	-

## Data Availability

The original contributions presented in this study are included in the article. Further inquiries can be directed to the corresponding author.
